# Effectiveness of Transcutaneous Electrical Nerve Stimulation with Taping for Stroke Rehabilitation

**DOI:** 10.1155/2021/9912094

**Published:** 2021-08-25

**Authors:** Tae-Sung In, Jin-Hwa Jung, Kyoung-Sim Jung, Hwi-Young Cho

**Affiliations:** ^1^Department of Physical Therapy, Gimcheon University, Gimcheon, Republic of Korea; ^2^Department of Occupational Therapy, Semyung University, Jecheon, Republic of Korea; ^3^Department of Physical Therapy, Gachon University, Incheon, Republic of Korea

## Abstract

**Background:**

Spasticity is a factor that impairs the independent functional ability of stroke patients, and noninvasive methods such as electrical stimulation or taping have been reported to have antispastic effects. The purpose of this study was to investigate the effects of transcutaneous electrical nerve stimulation (TENS) combined with taping on spasticity, muscle strength, and gait ability in stroke patients.

**Methods:**

From July to October 2020, 46 stroke patients with moderate spasticity in the plantar flexors participated and were randomly assigned to the TENS group (*n* = 23) and the TENS+taping group (*n* = 23). All subjects performed a total of 30 sessions of functional training for 30 min/session, 5 days/week, for 6 weeks. For therapeutic exercise, sit-to-standing, indoor walking, and stair walking were performed for 10 min each. In addition, all participants in both groups received TENS stimulation around the peroneal nerve for 30 min before performing functional training. In the TENS+taping group, taping was additionally applied to the feet, ankles, and shin area after TENS, and the taping was replaced once a day. The composite spasticity score and handheld dynamometer measurements were used to assess the intensity of spasticity and muscle strength, respectively. Gait ability was measured using a 10 m walk test.

**Results:**

The spasticity score and muscle strength were significantly improved in the TENS+taping group compared to those in the TENS group (*p* < 0.05). A significant improvement in gait speed was observed in the TENS+taping group relative to that in the TENS group (*p* < 0.05).

**Conclusions:**

Thus, TENS combined with taping may be useful in improving spasticity, muscle strength, and gait ability in stroke patients. Based on these results, an additional application of taping could be used to enhance the antispastic effect of TENS or other electrical stimulation treatments in the clinic. A long-term follow-up study is needed to determine whether the spasticity relieving effect persists after taping is removed.

## 1. Background

Stroke is a disease in which brain function is impaired due to a sudden interruption of blood supply to the brain tissue [1]. In stroke patients, the ability of the central nervous system to control the affected side is compromised, the coordination of agonist and antagonist muscles deteriorates, and proprioception and balance control are impaired [2]. In addition, it has been reported that 36%–70% of stroke patients experience spasticity [3, 4], which negatively impacts their functional recovery and results in poor quality of life [5, 6]. In particular, spastic hypertonia of the plantar flexor muscles can cause abnormal gait related to equinovarus foot deformity [7].

Various types of physical therapy interventions, antispastic drugs, and surgical interventions have been used to treat spasticity induced by stroke [8]. Physical therapy interventions include positioning training, stretching, thermotherapy, cryotherapy, facilitatory or inhibitory techniques of voluntary activity, hydrotherapy, vibratory stimulation, electrical stimulation, and acupuncture [9, 10]. Functional electrical stimulation (FES) and transcutaneous electrical nerve stimulation (TENS) are known to have antispastic effects on patients with certain neurological deficits. These interventions also have the advantage of being noninvasive, atraumatic, and easily applicable when compared to acupuncture. In a recent study, relief of spasticity in patients with spinal cord injury was observed for 4 h following 30 min of FES and TENS application, and it was reported that there was no significant difference between the two methods [11]. However, in the case of FES, there is insufficient evidence regarding its ability to reduce spasticity, especially in patients with stroke [9, 12, 13]. Recently, many studies have reported that TENS [14, 15] and taping [16–18] are effective interventions for the management of spasticity associated with neurological disorders. TENS is known to regulate spasticity through various mechanisms, such as by increasing presynaptic inhibition or by reducing the excitability of stretch reflexes [19]. According to a meta-analysis study of the effects of TENS on spasticity, TENS application over nerve or muscle belly in stroke patients for more than 30 min had a strong therapeutic effect on improving spasticity [20]. Tinazzi et al. demonstrated that corticomotor excitability of the area to which TENS was applied was reduced, suggesting that electrical stimulation applied to the somatic area may affect and regulate brain plasticity [21]. However, the application time of electrotherapy is approximately only 30 min/day. As the expression of titin and collagen is different in spastic muscles [22], maintaining the muscle length in a shortened state can change the microstructure over a short period of time [23, 24]. Therefore, additional therapeutic treatments are needed to further enhance and maintain the antispastic effects of TENS.

Recently, other treatments such as stretching, casting, and taping have been applied to enhance the effect of botulinum toxin on reducing spasticity, and taping has been reported to be more effective than electrotherapy or stretching [17]. In a recent study that applied taping to increase the efficacy and effect duration of botulinum toxin injection, it was reported that taping was more effective than stretching exercises. The authors suggested that taping could strengthen the internalization of botulinum toxin type A as it continuously stretches the muscle, thereby exerting a positive effect on the viscoelastic properties of spastic muscles [25].

To date, several studies have reported the synergistic effects of taping and botulinum toxin; however, whether or not taping could enhance the antispastic effect of TENS has not been studied. Therefore, this study is aimed at investigating the effects of TENS combined with taping on spasticity, muscle strength, and gait ability in stroke patients. We applied TENS and functional training to both the experimental and control groups to confirm the antispastic effect of TENS, and the experimental group additionally received taping. We hypothesized that the TENS application reduces spasticity, while the additional application of taping around the ankle joint further enhances the antispastic effect and improves muscle strength and gait ability in stroke patients.

## 2. Material and Methods

### 2.1. Study Design and Setting

This study was designed as a single-blind randomized controlled trial and was conducted at a specialized rehabilitation center for inpatients with neurological disorders.

### 2.2. Participants

This study was conducted on 50 stroke patients admitted to M Hospital in Gyeonggi-do, Korea, from July to October 2020. The inclusion criteria were as follows: (1) a diagnosis of stroke, (2) first episode of unilateral stroke with hemiparesis caused by hemicerebral damage, (3) ability to communicate, (5) ability to walk 10 min, (6) composite spasticity score of ≥10 in the affected plantar flexors, (7) no hemispatial neglect or sensory deficit, and (8) no contraindications to TENS [26–28]. The general characteristics of the participants are presented in Table 1. All participants voluntarily provided informed consent before participating in this study, which was approved by the Institutional Review Board of Gachon University (approval no. 1044396-202006-HR-113-01).

### 2.3. Experimental Procedure

The sample size was calculated using the G∗power 3.1.9.4 software (Heinrich-Heine-University Düsseldorf, version 3.1.9.4, Düsseldorf, Germany), and the mean power and alpha error of this study were set to 0.8 and 0.05, respectively. Based on a pilot study of 12 participants, the effect size was set to 0.81. As a result, the acceptable number of participants in each group was 20. To recruit participants, guidance documents for recruitment of research subjects were attached to M Hospital located in Gyeonggi-do, Republic of Korea. Fifty-five patients with chronic stroke volunteered to participate in the study. Five patients were excluded for the following reasons: three patients did not meet the selection criteria and two patients failed to follow the protocol of the study due to personal reasons. Participants were randomly assigned to either the TENS+taping group (*n* = 25) or the TENS group (*n* = 25) in a 1 : 1 ratio via sealed envelopes containing an assignment code. No differences in outcome measures were detected at baseline between the groups (paired *t*-test, all *p* > 0.05). Intervention allocation was recorded in a password-protected document to maintain blinding. All data were measured by the same blinded physical therapist before the intervention began and at the end of the 6-week intervention period.

### 2.4. Intervention

Both the TENS+taping and TENS groups performed sit-to-stand exercise, indoor walking, and stair walking for 10 min each, for a total of 30 min. The sit-to-stand exercise started with the participants sitting on a height-adjustable table, the knee joint flexed at 90°, and the feet placed 10 cm behind the knee. For indoor walking, straight walking and S-shaped walking were performed for 10 min. The participants climbed and descended 10 cm of stairs for stair walking training. Training was conducted five times a week for 6 weeks.

All subjects received TENS around the peroneal nerve for 30 min prior to functional training, following the method of Jung et al. [29] regarding the application of TENS. TENS was applied to the affected common peroneal nerve at twice the intensity of the sensory threshold, and a pulse width of 200 *μ*s was delivered at a frequency of 100 Hz. For the safety of the participants, the training was supervised by a physical therapist with at least 5 years of clinical experience.

In addition, taping was additionally applied to the TENS+taping group during the training period. The tape application followed Karadag-Saygi et al.'s method [30], in which the ankle was placed high in a neutral position and the tape was applied in four steps. First, in the supine position, the tape was stretched to approximately 120% of the maximum length and attached to the tibialis anterior from the middle of the dorsal metatarsal bone to the lower part of the fibular bone head. Second, two strips of tape were attached from the heel to the medial and lateral heads of the calf muscles. Third, the tape was attached to the medial and lateral ankles starting from the arch of the sole of the foot. Fourth, the tape was attached to both the malleoli across the front of the ankle joint. The tape was then replaced daily. When severe redness, blisters, or discomfort occurred, the training was discontinued.

### 2.5. Outcome Measure

The primary outcome measure in this study was spasticity, and the secondary outcome measures were muscle strength and gait ability.

The composite spasticity score (CSS) is an evaluation tool for measuring the intensity of spasticity in the plantar flexors and is composed of the Achilles tendon jerk, passive ankle dorsiflexion, and ankle clonus [31]. A total score of ≤9 indicates mild spasticity, 10-12 indicates moderate spasticity, and 13-16 indicates severe spasticity [32].

A handheld dynamometer (model 01163; Lafayette Inc., Lafayette, IN, USA) was used to measure the strength of the plantar flexor and knee extensor muscles. To measure knee extensor strength, the participants were instructed to straighten the knee against the dynamometer while sitting on a chair, so that the knee joint was in 90° flexion. To measure the strength of the plantar flexors, the hip and knee joints were extended while the participants were lying on a mat to perform plantar flexion against resistance. Muscle strength was measured three times, and the average value was used for the analysis. This measurement method has proven reliable in patients with neurological disorders [33].

Gait ability was measured using a 10 m walk test. This test measures the time it takes the subject to walk 10 meters and has been previously reported to have high intrarater reliability and interrater reliability (*r* = 0.89-1.00) [34].

### 2.6. Data Analysis

SPSS Statistics Version 21.0 (IBM Corp., Armonk, NY, USA) was used for the statistical analysis. The Shapiro-Wilk test was used to evaluate the normality of the variables. An independent *t*-test and chi-squared test were used to compare the baseline characteristics of the two groups of continuous and categorical variables. A paired *t*-test was performed to examine the changes within a group in terms of spasticity, strength, and gait speed. An independent *t*-test was used to determine whether significant differences existed between the two groups in the degree of change in spasticity, strength, and gait speed before and after 6 weeks of training. The significance level was set at *p* < 0.05.

## 3. Results

**Figure 1:**
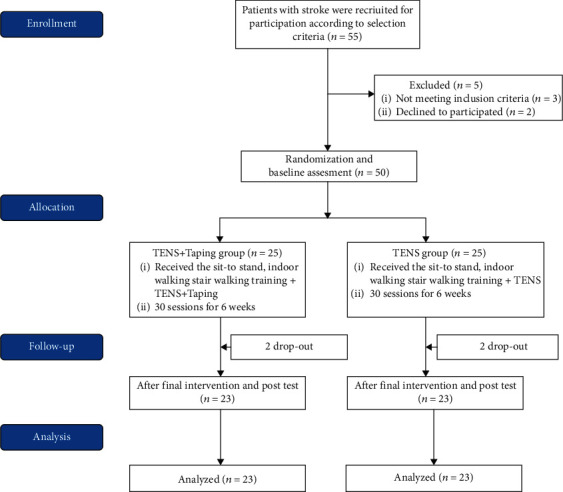
Flow diagram of participants.

During training, four participants dropped out because of a change in address and the development of skin redness and blisters, and 46 participants were evaluated after training (Figure 1). No significant differences were found in any variables of general characteristics between the TENS+taping and TENS groups at baseline (Table 1).

After training, both the TENS+taping and TENS groups showed a significant decrease in spasticity (*p* < 0.05). Additionally, the TENS+taping group (12.1 ± 2.1 score vs. 8.7 ± 1.9 score, *p* < 0.05, effect size (ES): 1.69) showed a greater degree of improvement in spasticity than the TENS group (12.4 ± 2.5 score vs. 11.1 ± 2.0 score, *p* < 0.05, ES: 0.57).

The TENS+taping and TENS groups showed a significant increase in knee extensor and plantar flexor muscle strength after the intervention (*p* < 0.05), but a significantly greater improvement was observed in the TENS+taping group (each 11.2 ± 1.9 kg vs. 14.2 ± 1.7 kg, *p* < 0.05, ES: 1.66 and 12.0 ± 2.3 kg vs. 15.9 ± 2.6 kg, *p* < 0.05, ES: 1.58) than in the TENS group (each 10.6 ± 2.4 kg vs. 12.3 ± 2.4 kg, *p* < 0.05, ES: 0.71 and 12.4 ± 2.0 kg vs. 13.7 ± 1.8 kg, *p* < 0.05, ES: 0.68).

Both groups showed a significant improvement in gait speed after the intervention compared to before the intervention (*p* < 0.05). At the posttest, the TENS+taping group (25.2 ± 4.2 s vs. 20.1 ± 2.5 s, *p* < 0.05, ES: 1.39) showed a more significant improvement in gait speed than the TENS group (25.9 ± 4.6 s vs. 23.6 ± 4.0 s, *p* < 0.05, ES: 0.53) (Table 2).

We instructed subjects and therapists participating in the study to report adverse effects such as redness, itching, pain, abnormal sensations, and other dermatitis caused by the application of TENS and taping. Fortunately, no subjects reported adverse events during the study period. This suggests that TENS and taping could be effectively used in the clinic to improve spasticity in stroke patients without the risk of side effects.

## 4. Discussion

This study investigated the effectiveness of the combination of TENS and taping in stroke patients with regard to spasticity in the plantar flexor muscles. As a result of this study, both groups to which TENS was applied showed a significant reduction in spasticity of the plantar flexors. A systematic review study of nonpharmacological interventions for spasticity management provided moderate evidence for electrical stimulation and acupuncture as adjunct therapy, and low-level evidence was reported for rehabilitation programs aimed at relieving spasticity, such as movement therapy, stretching, splinting, and occupational therapy [35]. TENS is known to modulate brain plasticity by reducing corticomotor excitability of the stimulated somatosensory regions [21], has fewer side effects than other therapeutic interventions for spasticity, and can be self-administered [36]. In addition, in a meta-analysis study on the management of spasticity by TENS, strong evidence was found that TENS reduces spasticity of the lower extremity in stroke patients and that TENS is particularly effective when combined with other treatments [20]. The application time of TENS is 30 min/day, which is a very small proportion of the whole day. Studies investigating the protein properties of spastic muscles have indicated that the microstructure can change in a short time if the muscle length is kept in a shortened state [23, 24]. Therefore, in this study, taping was additionally applied to the spastic plantar flexor muscles. As a result, the TENS+taping group showed a significant reduction in spasticity compared to the TENS group without taping. In a study by Carda et al., three additional interventions (casting, stretching, and taping) were applied for a week to enhance the spasticity-relieving effect of botulinum toxin type A. The study results showed that spasticity and gait ability improved only after casting or taping and that the relief effect continued for up to 3 months after the application. They explained that the disparity in this result was caused by the difference in the application time, and, unlike stretching applied for 1 h, casting or taping applied for 24 h could keep the muscles stretched [25]. In addition, according to a meta-analysis study, taping is effective in relieving leg spasticity in stroke patients [37] and causes autogenic inhibition of hypertonic muscles as the muscles continue to stretch [38]. Reiter et al. reported that taping combined with low doses of botulinum toxin type A had the same effect as the injection at higher doses [16]. In this study, the TENS+taping group showed a significant improvement compared to the TENS group, which may be because taping applied to the calf muscle in an overexcited state may have reduced the cortical excitability and may provide stability to the unstable ankle joint in stroke patients. In addition, taping applied to the paralyzed tibialis anterior muscle stimulated proprioceptive sensory input to the muscles and soft tissues around the joint at the application site, suggesting that it had a positive effect on improving motor function.

In our results, the TENS+taping group (28.1%) showed a significant improvement in muscle strength relative to the TENS group (10.5%). Previous studies have reported that taping improves muscle strength by activating skin receptors or promoting muscle activity through an increase in blood flow; however, the effects of taping on improvement in muscle strength remain controversial [39]. Therefore, it is believed that the significant improvement in muscle strength in the TENS+taping group in this study was due to the decrease in spasticity, which could promote more accurate exercise and symmetrical weight support as the range of motion of the ankle joint was increased during the various functional training exercises. If spasticity occurs in the plantar flexors, the base of support decreases in the standing position and it becomes difficult to use ankle strategy [40, 41]. In a study on ankle-foot orthosis in stroke patients, it was reported that the use of orthosis improved the symmetry of weight-bearing and postural fluctuations in both legs [42]. Furthermore, Park et al. reported that taping improves the function of the ankle joint by supporting its movement rather than fixing the ankle joint like a brace [43]. An interesting finding in the study was the strengthening of the ankle plantar flexors, where spasticity was reduced by the application of taping. Spasticity is a condition that causes continuous muscle contractions in a specific muscle, which then interferes with the activation of agonist and synergistic muscles required to properly perform a motion. Muscle stiffness and tonus reduced by the taping application provide an environment that can strengthen the sliding interaction between the actin and myosin filaments in the corresponding muscle, which is thought to induce stronger muscle contraction.

In gait speed after training, the TENS+taping group showed a more significant increase than the TENS group. The hip and ankle joints play important roles in controlling the balance of physical stability. Among the parts of the human body, the ankle joints and feet have the primary functions of adjusting balance for postural sway, absorbing shock while walking, and providing propulsion to the lower limbs. For these functions, sufficient ankle joint range of motion, muscle strength, and proprioceptive sensation are required [44]. Therefore, it is believed that the TENS+taping group in this study showed a more significant improvement in gait speed owing to the increased range of motion and improved lower-extremity muscle strength resulting from the relaxation of spasticity in the plantar flexors. Furthermore, another reason for the improvement in gait speed may be the improvement in proprioception. Bischoff et al. reported that the application of taping improved proprioception through tactile stimulation, resulting in improved gait patterns including gait speed [45]. Although this study did not confirm the improvement in proprioception, it was considered that the improvement in postural control ability due to the improved proprioception contributed to the increase in walking speed.

In this study, TENS combined with taping was applied to stroke patients with plantar flexor spasticity, and the effects on spasticity, muscle strength, and gait ability were examined. All variables were significantly improved in the TENS+taping group compared to those in the TENS group. This is the first study to investigate the synergy effect of TENS and taping on spasticity, muscle strength, and gait function in stroke patients with plantar flexor spasticity. However, this study has several limitations. First, this study cannot generalize our findings due to the small sample size. Second, we did not include a control group to which placebo-taping was applied in the study, so we could not exclude the placebo effect of taping. Third, we used CSS consisting of more evaluation items than the Ashworth scale or modified Ashworth scale to measure stroke patients' spasticity more accurately. Although this method measures spasticity through the evaluation of three movements, it is a measurement method based on the subjective judgment of the assessor and also based on the score. Therefore, it will be necessary to perform more objective and more diverse evaluations using digital devices in the future. Fourth, we did not measure changes in proprioception or other sensory-motor systems that are associated with improvements in gait and muscle strength. Fifth, taping has various attachment methods due to differences in the intensity of tension, direction, and tape shape, and the effect may be different due to these differences. This study could not clearly elucidate these differences. Finally, although we changed the taping to the subject once a day, the subject was taped on the same site for 6 weeks. As a result, some subjects were withdrawn from the study due to redness and itchiness. Therefore, it is necessary to investigate whether the spasticity-relieving effect occurs even with an application time of <6 weeks and determine how long the effect persists after the removal of tape.

## 5. Conclusion

We demonstrated that TENS reduced spasticity and improved muscle strength and gait ability and that the additional application of taping significantly enhanced these effects. Taping is commonly used in clinical practice for pain relief, posture correction, and prevention of soft tissue damage in patients with musculoskeletal disorders and sports injuries. We suggest that taping can be used in clinical practice to improve spasticity, muscle strength, and gait ability in stroke patients through our results. Taping can be attached to muscles for long periods to provide continuous sensory input, effectively maintaining and enhancing the antispastic effect and improving motor function following electrotherapy and other short-term interventions. In future studies, it is necessary to investigate the duration of the antispastic effect following removal of taping, as well as identify a more effective taping application method following electrical stimulation to reduce spasticity.

## Figures and Tables

**Table 1:** Common and clinical characteristics of the subjects (*N* = 46).

Variables	TENS+taping group (*n* = 23)	TENS group (*n* = 23)	*p*
Sex (male/female)	15/8	16/7	0.753^b^
Affected side (right/left)	12/11	11/12	0.768^b^
Age (years)	53.7 ± 9.6^a^	54.4 ± 9.9	0.556^c^
Height (cm)	165.8 ± 9.2	166.1 ± 9.3	0.799^c^
Weight (kg)	62.4 ± 8.7	63.9 ± 8.4	0.522^c^
Stroke duration (months)	7.9 ± 2.0	7.1 ± 2.6	0.256^c^

^a^Mean ± standard deviation; ^b^chi-squared test; ^c^independent *t*-test.

**Table 2:** Subject scores before and after intervention.

Test	Group	CSS score (spasticity)	Muscle strength (kg)		Gait speed (s)
			Knee extensor	Ankle plantar flexor	
Pre	TENS+taping group	12.1 ± 2.1	11.2 ± 1.9	12.0 ± 2.3	25.2 ± 4.2
	TENS group	12.4 ± 2.5	10.6 ± 2.4	12.4 ± 2.0	25.9 ± 4.6
Post	TENS+taping group	8.7 ± 1.9	14.2 ± 1.7	15.9 ± 2.6	20.1 ± 2.5
	TENS group	11.1 ± 2.0	12.3 ± 2.4	13.7 ± 1.8	23.6 ± 4.0
Mean difference (95% CI)	TENS+taping group	-3.3 (-4.0~-2.7)^∗^	3.0 (2.4~3.5)^∗^	3.9 (3.1~4.7)^∗^	-5.0 (-6.2~-3.7)^∗^
	TENS group	-1.3 (-2.4~-0.2)^∗^	1.7 (1.1~2.3)^∗^	1.3 (0.2~2.4)^∗^	-2.3 (-3.4~-1.1)^∗^
*t*(*p*)		-3.388 (0.001)	3.298 (0.002)	3.972 (<0.001)	-3.598 (0.001)

Values are expressed as the mean ± standard deviation (SD). ^∗^Significant differences between pre- and posttests (*p* < 0.05). CSS: composite spasticity score; CI: confidence interval.

## Data Availability

The data of this study are available from the corresponding authors upon reasonable request.
