# Corrigendum to “Effectiveness of Transcutaneous Electrical Nerve Stimulation with Taping for Stroke Rehabilitation”

**DOI:** 10.1155/2022/9763093

**Published:** 2022-08-13

**Authors:** Tae-Sung In, Jin-Hwa Jung, Kyoung-Sim Jung, Hwi-Young Cho

**Affiliations:** ^1^Department of Physical Therapy, Gimcheon University, Gimcheon, Republic of Korea; ^2^Department of Occupational Therapy, Semyung University, Jecheon, Republic of Korea; ^3^Department of Physical Therapy, Gachon University, Incheon, Republic of Korea

In the article titled “Effectiveness of Transcutaneous Electrical Nerve Stimulation with Taping for Stroke Rehabilitation” [1], the authors wish to update the authors' contributions to the below:

“All authors have read and approved the final version of the manuscript. T.-s.I and J.-h.J are co-first authors. Conceptualization was performed by K.-s.J. and T.-s.I.; writing (original draft preparation) was performed by T.-s.I.; writing (review and editing) was performed by K.-s.J. and H.-y.C.; methodology was performed by H.-y.C.; formal analysis was performed by K.-s.J.; investigation was performed by J.-h.J.; measurement visualization was performed by J.-h.J.”
